# Randomised-controlled trial of a web-based dietary intervention for patients with type 2 diabetes mellitus: Study protocol of *my*DIDeA

**DOI:** 10.1186/1471-2458-11-359

**Published:** 2011-05-21

**Authors:** Amutha Ramadas, Kia Fatt Quek, Carina KY Chan, Brian Oldenburg, Zanariah Hussein

**Affiliations:** 1 Jeffrey Cheah School of Medicine and Health Sciences, Monash University Sunway Campus, 46150 Bandar Sunway, Petaling Jaya, Malaysia; 2 Department of Epidemiology and Preventive Medicine, Monash University Clayton Campus, Wellington Road, Clayton, Victoria 3800, Australia; 3 Department of Medicine, Hospital Putrajaya, Federal Government Administration Centre, Precinct 7, 62250 Putrajaya, Malaysia

## Abstract

**Background:**

The potential of web-based interventions in dietary behaviour modification of the diabetics has not been fully explored. We describe the protocol of a 12-month match-design randomised controlled trial of a web-based dietary intervention for type 2 diabetic patients with primary aim to evaluate the effect of the intervention on their dietary knowledge, attitude and behaviour (KAB). The secondary objective of this study is to improve the participants' dietary practices, physical measurements and biomarkers.

**Methods/Design:**

A minimum total sample of 82 Type 2 diabetics will be randomised, either to the control group, who will receive the standard diabetes care or the e-intervention group, who will participate in a 6-month web-based dietary intervention in addition to the standard care. The dietary recommendations are based on existing guidelines, but personalised according to the patients' Stages of Change (SOC). The participants will be followed up for 6 months post-intervention with data collection scheduled at baseline, 6-month and 12-month.

**Discussion:**

We are aiming for a net improvement in the KAB score in participants of the e-intervention group, besides investigating the impact of the e-intervention on the dietary practices, physical measurements and blood biomarkers of those patients. The successful outcome of this study can be a precursor for policy makers to initiate more rigorous promotion of such web-based programmes in the country.

**Trial registration:**

Clinicaltrials.gov NCT01246687

## Background

Diabetes is a growing concern in Malaysia and in the world. The prevalence of diabetes in adults worldwide was estimated to rise 366 million in the year 2030 from 171 million in 2000 [1]. The prevalence of Type 2 Diabetes Mellitus (T2DM) in Malaysians above 30 years old was reported to be between 11% and 14% in 2006, and it is estimated to rise further [2]. T2DM accounts for a huge burden of morbidity and mortality through micro and macrovascular complications [3,4]. This has lead to an increasing demand on dietary and lifestyle modifications to delay the disease progression.

Web-based interventions have been successfully implemented in improving self-management of diabetes [5-8], physical activity [9,10] and weight management [11,12] in adults with T2DM. A web-based nutrition education for diabetes prevention study among young adults has shown significant reduction in dietary fat intake [13]. To date there is no published study focused solely on dietary behaviour change in adults with T2DM via a website-based system. Nevertheless, dietary modification has been incorporated as a component of multifactorial behavioural interventions in diabetes prevention and management [11,12,14].

The success of a web-based intervention relies heavily on the website log-in rates, usability, and personalisation. Content development in simplified local language improves the usability of the website [15], while regular reinforcement using reminder services such as e-mails and SMS increases the log-in rates [16]. The use of behavioural theories could assist the personalisation of web-based interventions. Transtheoretical Model (TTM) [17] is one of such behavioural model that has been recently used in web-based interventions, especially those focusing on improving dietary and lifestyle behaviours [10,18].

The study protocol for a randomised controlled trial (RCT) of a web-based dietary intervention for patients with T2DM in Malaysia is presented here. We hypothesised that the e-intervention group will show a greater improvement in dietary KAB than the control group. Secondly, we hypothesised that the web-based intervention to results in a better dietary practices, anthropometric measurements and blood biomarkers in the e-intervention group than the controls.

## Methods/Design

### Study design

**Figure 1:**
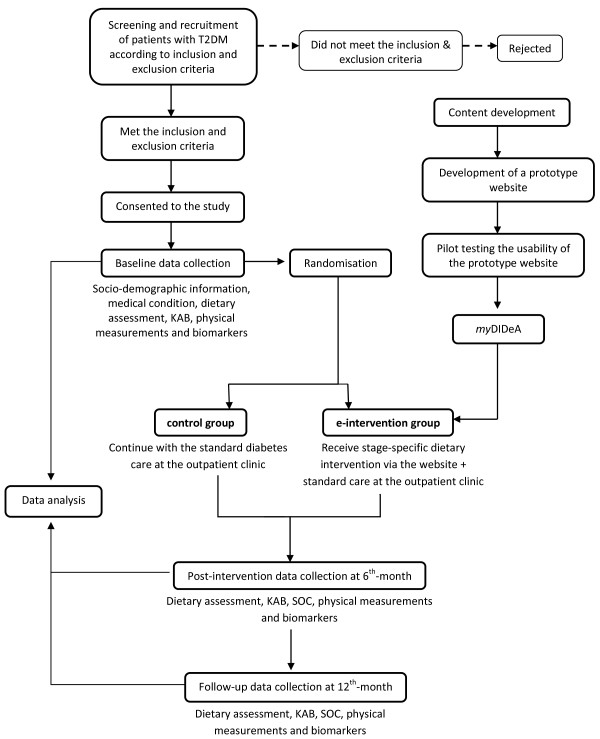
**Flow chart of the proposed study**.

This is a two-armed matched-design randomized controlled trial (RCT) scheduled for 12 months (Figure 1). The study is designed according to the recommendations of the CONSORT statement for randomised trials of non-pharmacologic treatment [19].

**Figure 2:**
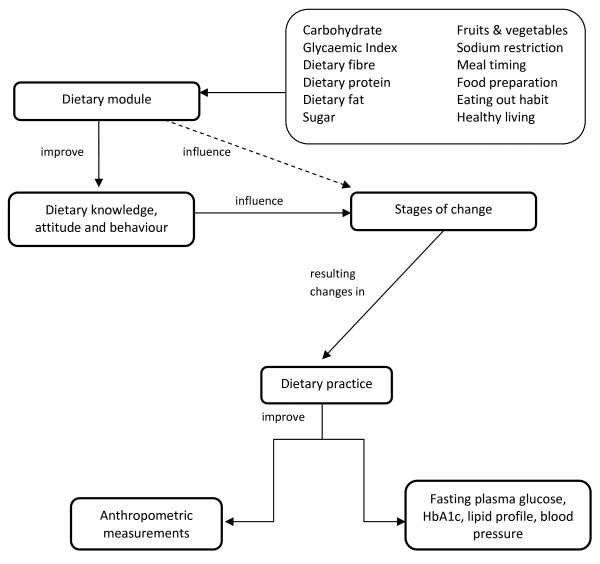
**Conceptual framework**.

The conceptual framework of the study is presented in Figure 2. Twelve dietary modules in the intervention package is personalised according to the patients' Stages of Change (SOC), and is expected to improve their dietary KAB and assist them to progress in their respective SOC. The improvements in KAB and progress in SOC are expected to be reflected in the patients' dietary practices. Adoption of healthy dietary practices then is expected to be reflected on the anthropometric measurements and biomarkers.

### Study aims

The primary aim of our study is to evaluate the effect of a six month dietary e-intervention on dietary knowledge, attitude and behaviour (KAB) in patients with T2DM.

The secondary aim is to determine the impact of the intervention on dietary practices (nutrient intake, dietary GI, food frequency score, supplements intake, cooking method and eating out habit), physical measurements (height, weight, body mass index, waist circumference, body fat percentage and blood pressure) and blood biomarkers (fasting blood glucose (FBG), glycosylated haemoglobin (HbA1c) and lipid profile).

### Ethics approval

The study has received ethics approval from Malaysian Ethics Research (NMRR-09-303-3416) and Monash University Human Research Ethics Committee (CF09/1583 - 2009000877).

### Study sample

#### Sample size and power calculation

The sample size calculation is based on a difference in fat and fibre-related behaviour score as reported by a previous computer assisted dietary intervention study in type 2 diabetics [20]. Using a reduction in the mean behaviour score from 1.93 ± 0.5 (baseline) to 1.69 ± 0.4 (post intervention), the GPower software [21] calculated that 31 patients are needed in each group to detect this difference with a two-sided alpha of 0.05, a power of 80%. Based on 30% attrition rate for one year, a minimum of 41 participants are required in each group.

#### Recruitment process

The eligibility screening, recruitment of study participants and data collections are conducted in the outpatient medical clinic of three urban hospitals in Malaysia. Diabetic nurses provide assistance in identifying potential study participants according to the eligibility criteria (Table 1). If the patients are found have potential to participate in the study, they complete the baseline questionnaire after giving a written informed consent. They are, however, excluded if the baseline dietary KAB score was more than 50%.

**Table 1:** Eligibility criteria

Inclusion	Exclusion
Mentally sound men and women who are ≥ 18 years old. Literate with a fair command of English and/or Malay languages. Have access to the Internet at home, work or public place. Willing to access the study website at least once every fortnight. Have been confirmed of having HbA1c of ≥7.0%.	Pregnant, lactating or intend to become pregnant during the study period. Diagnosed with Type 1 Diabetes Mellitus (T1DM) or Gestational Diabetes Mellitus (GDM) Weighing more than 150% of the desired weight for height. Any pre-existing condition compromising the quality of life or ability to participate according to protocol. Have severe complications (chronic heart disease, cerebrovascular disease, diagnosed HIV/AIDS, cancer, emphysema, chronic liver or kidney disease) that would affect the subjects' ability to follow the tailored advice. Enrolled in other clinical studies. Having dietary KAB score more than 50% at baseline.

### Randomisation and treatment allocation

Eligible diabetic patients who have consented to participate are matched for age, sex and ethnicity, and randomised to either e-intervention or control group.

#### Control group

This group receives the usual standard treatment (diabetic control and management) given to patients with T2DM. Although the controls have access to the Internet, they receive neither website log-in information, nor any reinforcement via e-mail or SMS.

#### e-Intervention group

This group receives an intensive dietary intervention through the study website, personalised according to the participants' SOC, in addition to the usual standard treatment at the outpatient clinics.

### Intervention programme

#### Dietary module

Twelve dietary lesson plans are developed for the intervention based on the Nutrition Recommendations and Interventions for Diabetes by American Diabetes Association [22], Malaysian Clinical Practice Guidelines for Type 2 Diabetes Mellitus [23] and Malaysian Medical Nutrition Therapy Guidelines for Type 2 Diabetes [24]. The intervention's structure and materials are developed using TTM's Stages of Change construct [17] (Table 2). The content of each lesson plan is studied for its relevance to the local community and fine-tuned to suit local context.

**Table 2:** Five Stages of Change, its characteristics and relevant strategies (Kendra C 2010)

Stage	Characteristics	Strategies
**Pre-contemplation**	• Not intending to change in the next 6 months. •In denial or ignorant of the problem.	•Encourage the patient to self-analyse and rethink his/her behaviour. •Explain the risks of current behaviour.

**Contemplation**	•Intending to change in the next 6 months. •Having conflicting emotion.	•Weigh the pros and cons of changing behaviour. •Address barriers and encourage confidence.

**Preparation**	•Planning to change in the next 30 days and have made a previous attempt to improve. •Experimenting and collecting information about change.	•Prepare an action plan or goal. •Motivate the patient to change.

**Action**	•Taking actions towards achieving the goal for at least the last 6 months.	•Encourage the patient to seek social support and motivate him/her to sustain the behaviour. •Reward for the success.

**Maintenance**	•New behaviour is sustained for at least 6 months.	•Strategies to cope with temptation. •Reward for the success.

### Website design

**Figure 3:**
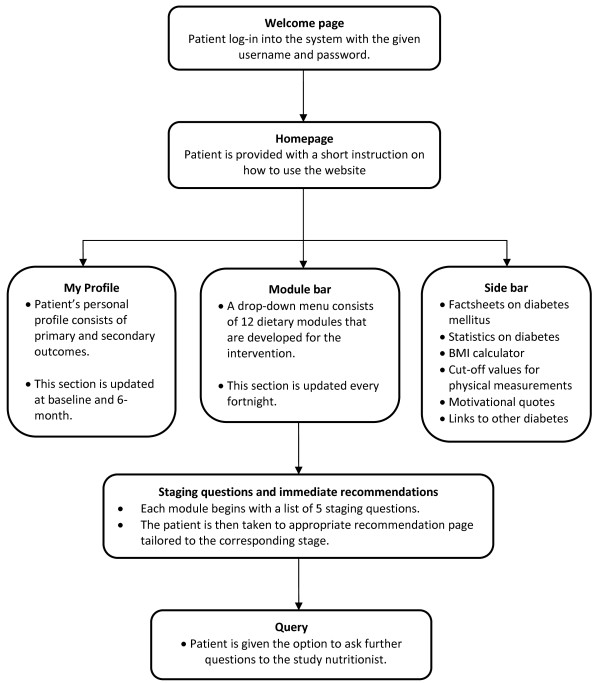
**The website structure**.

Suitable features to be highlighted in the website are discussed by a research panel comprising of nutritionist, behavioural psychologists, endocrinologist, public health expert, and a web master. Figure 3 outlines the structure of the study website, *my*DIDeA *(Dietary Intervention for Type 2 Diabetes Patients: An e-Approach)*.

*my*DIDeA will be tailored according to the participants' SOC. Each lesson plan has five Likert scale items (strongly agree = 5 to strongly disagree = 1) and participants are assigned to recommendations based on the score obtained. The recommendations are aimed to address the barriers and motivate the participants according to the lesson plan and stage. Relevant photographs and illustrations are added to enhance the understandability of the lesson plans. Factsheets and statistics on diabetes, links to existing diabetes resources and an example of ideal health profile also are included.

The participants are briefed on *my*DIDeA, and are provided with unique username and password via e-mail or SMS after the randomisation. Log-in reminders are sent to their e-mail each time *my*DIDeA is updated with new lesson plan. The participants are also encouraged to send their queries to the study nutritionist via the website.

### Outcome assessments

All assessments (Table 3) are conducted at the clinical settings by trained enumerators.

**Table 3:** Assesments that will be carried out in the study

Outcome	Measure	Instrument
Primary	Nutrition KAB related to T2DM	Dietary Knowledge, Attitude and Behaviour Questionnaire*

Secondary	Dietary practices Height Weight and body composition Waist circumference Systolic and diastolic blood pressure FBS, HbA1c and lipid profile	Semi-Food Frequency Questionnaire* 24-hour dietary recall Supplement intake Cooking method Eating out habit Body meter Four-point digital weighing scale Non-elastic measuring tape Automatic blood pressure monitor Medical record

Others/Covariates	Socio-demographic characteristics Medical history Physical activity Stages of change	Pretested questionnaire International Physical Activity Questionnaire Stages of Change Questionnaire*

*validated instrument

#### Primary outcomes

The primary outcome is assessed using a validated interviewer-administered dietary KAB questionnaire in English and Bahasa Malaysia.

#### Secondary outcomes

A semi-food frequency questionnaire (SFFQ) is used to record the subjects' dietary intake, while two days 24-hour dietary recall is used to analyse the nutrient intake [25]. Besides SFFQ and 24-hour dietary recall, consumption of supplements, cooking techniques and eating out habit for the past one month are also recorded. Anthropometric measurements (body mass index, waist circumference and percentage of body fat) and blood pressure are taken during the interview sessions. The results of participants' blood biomarkers are obtained from their medical record.

#### Process evaluation

Adherence to the intervention is assessed by the number of log-ins and duration spent in the website. Besides, the participants' satisfaction of the intervention is assessed by self-administered questionnaire at post-intervention.

#### Other measurements

The socio-demographic characteristics, medical history, and smoking and drinking habits are recorded in a structured questionnaire. International Physical Activity Questionnaire (IPAQ) is used to determine the level of physical activity of the participants [26].

### Blinding

It is not possible to blind study nutritionist, webmaster and the participants. The enumerators trained to collect the data, however, are blinded during the data collection.

### Statistical analysis

Axxya Systems Nutritionist Pro™ Diet Analysis is used to analyze the nutrient intakes and all statistical analyses are performed with IBM^® ^SPSS^® ^Statistics 17.0. Independent *t*-test or equivalent is used to determine differences between the study groups for continuous variables, while χ^2 ^or equivalent is used to determine the association between categorical variables. As this is a prospective RCT involving repeated measures, the ANOVA repeated measures model is applied to observe significant differences within the study groups. The evaluation of the intervention is based on an intention-to-treat analysis, with the *p *value 0.05 was taken as the level of significance.

## Discussion

This is a 12 months web-based dietary intervention RCT for patients with uncontrolled T2DM with the aim to improve diabetes-related dietary KAB. We are employing existing guidelines in the development of the intervention package that will be modified to suit local context. TTM's SOC construct will be incorporated in the development of the study website, *my*DIDeA. We are aiming for a net improvement in the KAB score in participants of the e-intervention group, besides investigating the impact of the e-intervention on the dietary practices, physical measurements and blood biomarkers of those patients.

We hope to capture the 'teachable moment' to promote dietary behaviour change in diabetics with uncontrolled HbA1c (≥7.0%) and lower level of diabetes-specific dietary KAB (≤50% of total score). We are anticipating an increase in dietary KAB at the end of the trial, which may contribute to a better blood glucose control and ultimately prevent complication due to diabetes.

Besides offering a theory and evidence based education program, this trial will be utilising bilingual educational materials that have been modified to suit the local content. Culturally sensitive dietary intervention [27] and web content [15] leave greater impact, giving this trial an edge in helping the patients to improve their dietary KAB and achieve better glycaemic control.

This trial is capable of generating a personalised dietary intervention program for a large group of patients. At the same time, the intervention program could be accessed 24 hours using any device connected to the Internet. This will empower the patients to have total control of the intervention materials.

We will be conducting this RCT in urban hospitals, which means the respondents may be of a higher socio-economic background than average Malaysians. This may not be representative of the entire population as uptake of internet is different in the urban as compared to the rural. However, we are expecting for a better response to a web-based program in the urban area and it will be interesting to investigate responses from those with higher socio-economic status to an interactive website. This trial can be a model that is first implemented at the urban area for us to evaluate its success. If successful, it can be a precursor for policy makers to initiate more rigorous promotion of such web-based programs to other parts of the country.

Like all web-based interventions, this trial's reach also depends on the participants' responsiveness. Besides personalisation according to SOC, culture and language, the success of this intervention relies on the log-in rates and usability of *my*DIDeA. In order to maximise the participation of the trial patients, we will be regularly reinforcing them to log-in via e-mail and SMS.

Although qualitative assessments such as in-depth interview or focus group discussion were not part of this RCT, the outcome evaluation is supplemented by a detailed process evaluation. The process evaluation is measuring program reach, dose delivered, dose received, fidelity and implementation as well as the participants'' satisfaction with the program and acceptability. Collectively, these measures will enable us to draw conclusions about potential program enhancers and barriers if the program were to be delivered in other settings and contexts.

## Competing interests

The authors declare that they have no competing interests.

## Authors' contributions

AR developed the study framework and will be involved in data collection and in the development and implementation of the intervention. QKF gave feedback on the research design and will be giving his expertise in data analysis and interpretation of the study results. CKYC and BO gave feedback on the study protocol and to provide expertise in the development of the intervention package. ZH is to provide expertise in selection of the study sample. All authors read and contributed to the final manuscript.

## Pre-publication history

The pre-publication history for this paper can be accessed here:

http://www.biomedcentral.com/1471-2458/11/359/prepub
